# The adverse effect of the COVID-19 pandemic on health service usage among patients with type 2 diabetes in North Karelia, Finland

**DOI:** 10.1186/s12913-022-08105-z

**Published:** 2022-06-01

**Authors:** Laura Inglin, Katja Wikström, Marja-Leena Lamidi, Tiina Laatikainen

**Affiliations:** 1 Institute of Public Health and Clinical Nutrition, University of Eastern Finland, Kuopio, Finland; 2 National Institute for Health and Welfare, Helsinki, Finland; 3 Joint municipal authority for North Karelia Health and Social Services (Siun sote), Joensuu, Finland

**Keywords:** Electronic health records, SARS-CoV-2, Coronavirus, Quality of care, Type 2 diabetes, Essential health services

## Abstract

**Aims:**

The COVID-19 pandemic has challenged health systems and their capacity to deliver essential health services while responding to COVID-19. This study examines the pandemic’s impact on health service usage among patients with type 2 diabetes in the North Karelia region, in Finland.

**Methods:**

This retrospective cohort study used electronic health records of 11,458 type 2 diabetes patients, comprising all primary and specialised care contacts in 2019 and 2020. We analysed diabetes and dental healthcare contacts to primary care nurses, doctors and dentists and all emergency visits in specialised care. We compared healthcare usage in three different periods in 2020 (pre-lockdown [1 January–15 March], lockdown [16 March–31 May], post-lockdown [1 June–31 December]) with the equivalent period in 2019.

**Results:**

During the lockdown period, the number of diabetes-related contacts decreased significantly but quickly increased again to nearly the same level as in 2019. Overall, healthcare usage was lower in the pandemic year, with proportionally 9% fewer contacts per person (mean 2.08 vs 2.29) and a proportionally 9% lower proportion of patients making any contact (59.9% vs 65.8%). The proportion of remote consultations was similar in both years in the pre-lockdown period (56.3–59.5%) but then increased to 88.0% during the 2020 lockdown. Patterns were similar when analysed by age group and gender. Emergency visits went down significantly at the beginning of the lockdown period, but a “rebound effect” was observed, so after the lockdown, the number of emergency visits in 2020 exceeded the numbers of the previous year.

**Conclusion:**

Despite the COVID-19 pandemic, diabetes care was continuous, and even elderly patients aged ≥70 years accessed the health services. The delivery of many essential services was facilitated by processes that strongly relied on telemedicine already before the pandemic.

**Supplementary Information:**

The online version contains supplementary material available at 10.1186/s12913-022-08105-z.

## Introduction

The outbreak of COVID-19 has challenged health systems and their ability to deliver essential health services while responding to the quickly evolving global pandemic. While social distancing measures effectively avoid the spread of COVID-19 [1], they conflict with comprehensive diabetes management, which requires regular follow-up visits to manage the disease and its complications. Simultaneously, older adults and people with diabetes have been identified to be at increased risk of mortality [2, 3] and severe infection from COVID-19 [3].

The European Observatory on Health Systems and Policies has predicted increased demand for health services (besides care for COVID-19 cases and long-COVID cases) from service backlogs and treatment disruptions, the physical and psychological health impact of social distancing measures, and the pandemics’ long-term economic consequences [4]. Many countries have reported severe disruptions to their regular service delivery, including essential health services, and especially dental services and the diagnosis and treatment of non-communicable diseases (NCDs) [5]. Several studies on the impacts of the pandemic on people with diabetes and diabetes services across Europe have revealed a severe decline in the level of provided diabetes care and care outcomes [6–9]. Sharp drops in emergency care appointments for conditions other than COVID-19 in the early phase of the pandemic were also observed [10–17].

In Finland, the government declared a state of emergency early on, imposing a national lockdown from 16 March until mid-May 2020, when restrictions were gradually loosened [18]. During the lockdown until mid-June, special recommendations were issued for residents aged 70 and over, urging them to stay at home and avoid unnecessary social contact. In the North Karelia region, the pandemic situation resulted in significant changes and restrictions to healthcare services and operations. These included cancelling non-urgent appointments, especially for high risk patients, expanding remote services and limiting on-site assessments to issues that could not be handled remotely [19, 20]. According to the FinSote National survey, 18% of scheduled appointments for individuals aged over 54 years were cancelled or postponed in North Karelia during the first six to 8 months of the pandemic [21].

Undoubtedly, the lockdown and restrictive measures affect the accessibility and organisation of services drastically [22]. However, the extent to which accumulated service needs may lead to a worsening of health problems and an increase in the need for assistance in the longer term vastly depends on the magnitude and duration of the service and treatment backlogs and how providers have managed to reduce them after spring 2020.

There is a lack of knowledge on the effects of COVID-19 on healthcare usage among type 2 diabetes patients beyond the first few months of the pandemic. Therefore, this study examines the impact of the pandemic on the usage of essential routine care and emergency care services by type 2 diabetes patients at different stages of the pandemic in the North Karelia region of Finland.

## Methods

### Study population

This retrospective cohort study used regional electronic health records (EHRs) from the Mediatri database of the Joint Municipal Authority for North Karelia Social and Health Services (Siun sote), covering all primary healthcare (PHC) and specialised healthcare (SHC) services in 2019 and 2020. Data were retrieved for all patients diagnosed with type 2 diabetes by the end of 2018 (ICD-10 code E11) who were alive by the end of 2020 and permanently lived in North Karelia (*n* = 11,458).

### Variables

From the EHRs we obtained information on age (by 1 January 2020), gender, and healthcare usage such as contact frequency, the reason for contact (ICD-10 or ICPC-2 code), occupational classification of the attending professional, urgency status, and type of contact. There was no missing data in the dataset. We analysed all contacts related to type 2 diabetes (ICD-10: E11; ICPC-2: T90) with nurses or doctors and all contacts related to dental care relevant for type 2 diabetes management (ICD-10: K02, K04–05; ICPC-2: D82) with dentists in PHC. We distinguished face-to-face appointments and remote consultations consisting mainly of phone calls but also a few video consultations. We identified all urgent contacts in SHC and analysed these emergency visits. We created two age groups based governmental special recommendation for the elderly population aged 70 years and over.

The study period in 2020 was divided according to the timing of the COVID-19 lockdown in Finland. The pre-lockdown period (1 January–15 March 2020) was defined as the period from the beginning of the year until the national lockdown. The lockdown period (16 March–31 May 2020) was defined as the period during which most social distancing measures were still in place before gradually being relaxed. The post-lockdown period (1 June–31 December 2020) was defined as the remaining time of the calendar year. The comparison periods in 2019 were divided similarly, taking the weekdays into account (pre-lockdown period: 1 January–17 March 2019; lockdown period: 18 March–2 June 2019; post-lockdown period: 3 June–31 December 2019).

The reasons for emergency visits were categorised using the Clinical Classifications Software Refined (CCSR) tool (version 2020.2; Healthcare Cost and Utilization Project) [23], which combines ICD-10 codes into over 530 clinically meaningful categories and 21 chapters. This was adapted to take country differences in coding into account. A custom category for confirmed COVID-19 diagnoses (U07.1) was added. Some ICD-10 codes are cross-classified into more than one CCSR category because one code can describe multiple conditions or a condition and a common symptom/manifestation. Therefore, the sum of all visits according to the CCRS categories exceeds the number of real encounters. The mean monthly visits per period were calculated as the total visits divided by the number of months (*n* = 12 months for year, *n* = 2.5 for pre-lockdown and lockdown period, *n* = 7 for post-lockdown period).

All research procedures were employed in accordance with the relevant guidelines and regulations.

### Statistical analyses

We used means and percentages to describe the number of contacts, the proportion of remote contacts among all contacts and the proportion of patients seeking healthcare services. To record differences between the years in different periods, proportional percentages were used in the tables and absolute numbers were used in the figures. We used logistic regression models for dichotomous outcomes and non-parametric Wilcoxon signed-rank tests for continuous outcomes to study the difference between the years. To analyse the difference in the changes in different age groups and according to gender, we used logistic regression models with interaction terms and a Mann-Whitney U-test to determine the difference between the years for each patient and period. Two-sided *P* values < 0.05 are statistically significant and are summarised in the Supplementary Tables 1 to 3. The statistics were done using R (4.0.5) [24] and the *CCS* package [25].

## Results

The mean age of the 11,458 type 2 diabetes patients included in this study was 69.4 years, and the proportion of women was 45.6%.

### Type 2 diabetes care in primary care

PHC service usage related to type 2 diabetes care was significantly lower in 2020 compared to 2019. The mean number of all contacts (appointments and remote consultations) per person decreased by 9.2% from 2.29 to 2.08 (*p* < 0.001) and proportion of patients with any contact decreased by 8.9% from 65.8 to 59.9% (*p* < 0.001) (Table 1). The mean number of contacts did not differ between those patients with at least one contact 2019 and those in 2020 (Supplementary Table 4). While there was no statistically significant difference between the year in the mean number for all contacts per person in the pre-lockdown periods (*p* = 331), it was 15.7% (p < 0.001) and 9.6% (p < 0.001) lower in the lockdown and post-lockdown periods in 2020 than in 2019, respectively (Table 1). The proportion of remote consultations was 55.6% in 2019 and 75.3% in 2020.

**Table 1:** The number of patients and contacts (appointments and remote consultations)^1^

	Year			Pre-lockdown^**2**^			Lockdown^**3**^			Post-lockdown^**4**^		
Cohort (***n*** = 11,458)	2019	2020	Change (in %)	2019	2020	Change (in %)	2019	2020	Change (in %)	2019	2020	Change (in %)
**Primary care T2D-related contacts (nurse/doctor)**												
N of contacts	26,226	23,817	- 9.2*	5736	5660	- 1.3	6055	5106	-15.7*	14,435	13,051	- 9.6*
N of contacts per person, mean [min-max]	2.29 [0–42]	2.08 [0–33]	- 9.2*	0.50 [0–17]	0.49 [0–17]	- 1.3	0.53 [0–17]	0.45 [0–13]	- 15.7*	1.26 [0–26]	1.14 [0–26]	- 9.6*
N of appointments per person, mean [min-max]	1.02 [0–41]	0.51 [0–13]	- 49.5*	0.22 [0–16]	0.20 [0–5]	- 9.2*	0.24 [0–10]	0.05 [0–5]	- 77.6*	0.56 [0–25]	0.26 [0–11]	- 53.2*
N of remote contact per person, mean [min-max]	1.27 [0–26]	1.57 [0–30]	+ 23.0*	0.28 [0–13]	0.30 [0–13]	+ 4.8	0.29 [0–13]	0.39 [0–13]	+ 35.7*	0.70 [0–18]	0.88 [0–25]	+ 25.1*
Proportion of patients with any contact, % (±SE)	65.8 (±0.4)	59.9 (±0.4)	- 8.9*	28.9 (±0.5)	27.7 (±0.4)	- 4.2*	30.2 [±0.4]	24.3 (±0.4)	- 19.3*	50.8 [±0.5]	44.2 (±0.7)	- 13.0*
Proportion of patients with appointments, % (±SE)	52.8 (±0.5)	33.4 (±0.4)	- 36.8*	18.4 (±0.5)	17.0 (±0.4)	- 7.6*	20.1 [±0.4]	5.1 (±0.2)	- 74.9*	37.9 [±0.5]	20.1 (±0.4)	- 46.8*
Proportion of patients with remote contact, % (±SE)	48.4 (±0.5)	52.5 (±0.4)	+ 8.3*	18.1 (±0.4)	18.6 (±0.4)	+ 2.7	18.4 [±0.4]	22.0 (±0.4)	+ 19.8*	34.2 [±0.4]	37.4 (±0.5)	+ 9.4*
Proportion of remote contacts among all contacts, % (±SE)	55.6 (±0.3)	75.3 (±0.3)	+ 35.4*	56.3 (±0.7)	59.8 (±0.7)	+ 6.2*	54.7 (±0.6)	88.0 (±0.5)	+ 60.9*	55.7 (±0.4)	77.1 (±0.4)	+ 38.4*
**Primary care T2D-related contacts with nurse**												
N of contacts per person, mean [min-max]	1.68 [0–42]	1.57 [0–27]	- 6.5*	0.37 [0–17]	0.37 [0–13]	- 0.5	0.38 [0–15]	0.35 [0–13]	- 9.9*	0.92 [0–26]	0.85 [0–20]	- 7.6*
Proportion of patients with any contact, % (±SE)	59.0 (±0.5)	53.8 (±0.5)	- 8.7*	24.3 (±0.4)	23.9 (±0.4)	- 1.7	25.3 (±0.4)	20.9 (±0.4)	- 17.5*	43.9 (±0.5)	38.5 (±0.5)	- 12.3*
Proportion of remote contacts among all contacts, % (±SE)	51.0 (±0.4)	72.5 (±0.3)	+ 42.2*	52.7 (±0.8)	54.6 (±0.8)	+ 3.6	48.4 (±0.8)	87.6 (±0.5)	+ 81.0*	51.4 (±0.5)	74.2 (±0.4)	+ 44.4*
**Primary care T2D-related contacts with doctor**												
N of contacts per person, mean [min-max]	0.61 [0–13]	0.51 [0–10]	- 16.5*	0.13 [0–5]	0.12 [0–4]	- 3.8	0.14 [0–4]	0.10 [0–5]	- 30.9*	0.34 [0–8]	0.29 [0–8]	- 15.0*
Proportion of patients with any contact, % (±SE)	38.1 (±0.5)	31.8 (±0.4)	- 16.5*	10.9 (±0.3)	10.3 (±0.3)	- 5.0	12.2 (±0.3)	8.5 (±0.3)	- 29.9*	24.4 (±0.4)	20.0 (±0.4)	- 18.0*
Proportion of remote contacts among all contacts, % (±SE)	68.2 (±0.6)	84.0 (±0.5)	+ 23.2*	67.0 (±1.2)	75.9 (±1.2)	+ 13.3*	71.3 (±1.1)	89.2 (±0.9)	+ 25.1*	67.4 (±0.8)	85.6 (±0.6)	+ 27.0*
**Primary care dental health appointments with dentists**												
N of appointments	5491	4578	- 16.6*	1236	1030	- 16.7*	1143	696	- 39.1*	3112	2852	- 8.4*
N of appointments per person, mean [min-max]	0.48 [0–13]	0.40 [0–11]	- 16.6*	0.11 [0–8]	0.09 [0–6]	- 16.7*	0.10 [0–5]	0.06 [0–4]	- 39.1*	0.27 [0–8]	0.25 [0–11]	- 8.4*
Proportion of patients with appointment, % (±SE)	21.2 (±0.4)	18.9 (±0.4)	- 10.9*	8.0 (±0.3)	7.2 (±0.2)	- 10.1*	7.7 (±0.3)	4.7 (±0.2)	- 38.9*	14.6 (±0.3)	13.7 (±0.3)	- 6.3*
**Specialised care emergency appointments**												
N of appointments	6660	6855	+ 2.9	1311	1471	+ 12.2*	1369	1176	- 14.1*	3980	4208	+ 5.7
N of appointments per person, mean [min-max]	0.58 [0–35]	0.60 [0–54]	+ 2.9	0.11 [0–6]	0.13 [0–15]	+ 12.2*	0.12 [0–10]	0.10 [0–13]	- 14.1*	0.35 [0–21]	0.37 [0–32]	+ 5.7
Proportion of patients with appointment, % (±SE)	27.5 (±0.4)	27.2 (±0.4)	- 0.9	8.1 (±0.3)	8.6 (±0.3)	+ 5.6*	8.2 (±0.23)	6.9 (±0.2)	- 16.1*	18.7 (±0.4)	18.9 (±0.4)	+ 1.2

*Abbreviations*: *SE* standard error

^1^Face-to-face appointments only in emergency and dental health care

^2^Pre-lockdown periods: 1 January–17 March 2019; 1 January–15 March 2020

^3^Lockdown periods: 18 March–2 June 2019; 16 March–31 May 2020

^4^Post-lockdown periods: 3 June–31 December 2019, 1 June–31 December 2020

* Statistically significant difference between 2019 and 2020 with p-value < 0.05 (Wilcoxon signed-rank test for the difference in continuous variables, logistic regression for proportions)

**Fig. 1:**
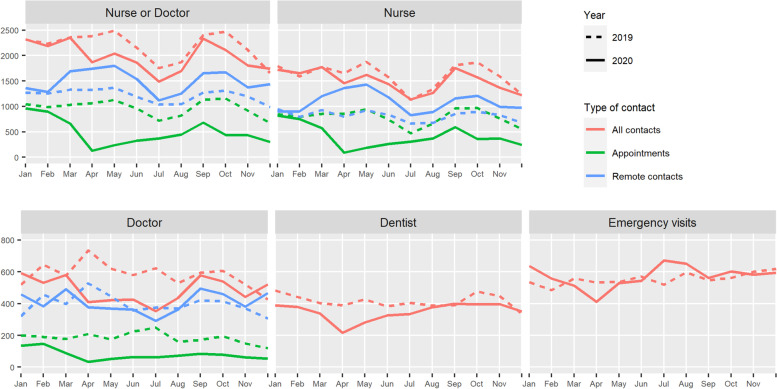
Monthly absolute health care contacts by contact type and year. Nurse/Doctor: All primary care contacts related to type 2 diabetes (ICD-10: E11; ICPC-2: T90) with nurse or doctor; Dentist: All primary care contacts related to dental care (ICD-10: K02, K04, K05; ICPC-2: D82) with a dentist (appointments only).; Emergency visits: All specialised emergency care contacts (appointments only)

In both years, contacts with doctors made up about a quarter of the contacts and were held remotely to an even greater degree compared to the contacts with nurses. While the mean number of appointments decreased in 2020 compared to 2019 in the pre-lockdown, lockdown and post-lockdown period by 9.2% (*p* = 0.001), 77.6% (p < 0.001) and 53.2% (p < 0.001), respectively, remote consultations increased by 4.8% (*p* = 0.156), 35.7% (*p* < 0.001), and 25.1% (p < 0.001), respectively. Figure 1 illustrates the simultaneous increase in remote consultations and decrease in appointments from March onwards.

### Dental care in primary care

The number of dental health contacts in PHC (appointments only) was lower in 2020 than in 2019, with a striking drop during the lockdown period (Fig. 1). In 2020, the mean number of appointments per person was 16.6% lower (0.40 vs 0.48, *p* < 0.001) and the proportion of patients with appointments was 10.9% lower (18.9% vs 21.2%, p < 0.001) than in 2019 (Table 2). Among patients with at least one appointment in 2020, the average number of appointments was also lower than among those in 2019 (Supplementary Table 4).

**Table 2:** The number of patients and contacts (appointments and remote consultations)^1^ by age group

	Year			Pre-lockdown^**2**^			Lockdown^**3**^			Post-lockdown^**4**^		
	2019	2020	Change (in %)	2019	2020	Change (in %)	2019	2020	Change (in %)	2019	2020	Change (in %)
**Under 70 years (*n*= 5435)**												
**Primary care T2D-related contacts (nurse/doctor)**												
N of contacts, mean [min-max]	2.29 [0–42]	2.13 [0–30]	- 7.2*	0.51 [0–17]	0.51 [0–17]	+ 0.2	0.53 [0–14]	0.46 [0–13]	- 12.0*	1.26 [0–26]	1.15 [0–26]	- 8.2*
Proportion of patients with any contact, % (±SE)	64.3 (±0.7)	59.4 (±0.7)	- 7.6*	29.1 (±0.6)	27.5 (±0.6)	- 5.6	29.3 (±0.6)	24.6 (±0.6)	- 15.9*	49.1 (±0.7)	43.1 (±0.7)	- 12.2*
Proportion of patients with appointments, % (±SE)	52.5 (±0.7)	34.4 (±0.6)	- 34.5*†	18.4 (±0.5)	17.6 (±0.5)	- 4.6	19.9 (±0.5)	5.9 (±0.3)	- 70.4*†	36.9 (±0.7)	19.9 (±0.5)	- 46.0*
Proportion of patients with remote contact, % (±SE)	47.2 (±0.7)	51.6 (±0.7)	+ 9.3*	18.6 (±0.5)	18.4 (±0.5)	- 1.5	17.9 (±0.5)	21.7 (±0.6)	+ 21.5*	33.1 (±0.6)	36.6 (±0.7)	+ 10.6*
Proportion of remote contacts among all contacts, % (±SE)	55.6 (±0.4)	75.2 (±0.4)	+ 35.3*	56.3 (±0.9)	59.7 (±0.9)	+ 6.0*	54.5 (±0.9)	86.6 (±0.7)	+ 58.9*†	55.8 (±0.6)	77.5 (±0.5)	+ 38.9*
**Primary care dental health appointments with dentists**												
N of appointments per person, mean [min-max]	0.64 [0–13]	0.54 [0–11]	- 15.0*	0.15 [0–8]	0.11 [0–6]	- 23.4*†	0.13 [0–5]	0.09 [0–4]	- 30.6*	0.35 [0–8]	0.33 [0–11]	- 5.6
Proportion of patients with appointment, % (±SE)	27.0 (±0.6)	23.9 (±0.6)	- 11.4*	10.7 (±0.4)	9.1 (±0.4)	- 15.4*	10.3 (±0.4)	7 (±0.4)	- 31.6*†	18.6 (±0.5)	17.7 (±0.5)	- 4.6
**Specialised care emergency appointments**												
N of appointments per person, mean [min-max]	0.50 [0–34]	0.49 [0–54]	-1.3	0.09 [0–6]	0.11 [0–15]	+ 15.4	0.10 [0–10]	0.08 [0–13]	- 19.7*	0.03 [0–21]	0.30 [0–32]	- 0.1
Proportion of patients with appointment, % (±SE)	23.1 (±0.6)	22.4 (±0.6)	- 3.0	6.7 (±0.3)	7.3 (±0.4)	+ 8.7	6.9 (±0.3)	5.4 (±0.3)	- 22.1*	15.6 (±0.5)	15.2 (±0.5)	- 2.8
**70+ years (*n*= 6023)**												
**Primary care T2D-related contacts (nurse/doctor)**												
N of contacts, mean [min-max]	2.28 [0–39]	2.03 [0–33]	- 11.0*	0.49 [0–14]	0.48 [0–13]	- 2.7	0.53 [0–17]	0.43 [0–12]	- 19.0*	1.26 [0–23]	1.13 [0–25]	- 10.8*
Proportion of patients with any contact, % (±SE)	67.1 (±0.6)	60.4 (±0.6)	- 10.0*	28.7 (±0.6)	27.9 (±0.6)	- 2.8	31.0 (±0.6)	24.1 (±0.6)	- 22.1*	52.3 (±0.6)	45.2 (±0.6)	- 13.7*
Proportion of patients with appointments, % (±SE)	53.1 (±0.6)	32.5 (±0.6)	- 38.8*†	18.4 (±0.5)	16.5 (±0.5)	- 10.4*	20.3 (±0.5)	4.3 (±0.3)	- 78.9*†	38.7 (±0.6)	20.3 (±0.5)	- 47.6*
Proportion of patients with remote contact, % (±SE)	49.5 (±0.6)	53.2 (±0.6)	+ 7.5*	17.6 (±0.5)	18.8 (±0.5)	+ 6.7	18.8 (±0.5)	22.3 (±0.5)	+ 18.3*	35.1 (±0.6)	38.1 (±0.6)	+ 8.4*
Proportion of remote contacts among all contacts, % (±SE)	55.6 (±0.4)	75.4 (±0.4)	+ 35.6*	56.3 (±0.9)	60.0 (±0.9)	+ 6.6*	54.8 (±0.9)	89.4 (±0.6)	+ 63.1*†	55.6 (±0.6)	76.7 (±0.5)	+ 37.9*
**Primary care dental health appointments with dentists**												
N of appointments per person, mean [min-max]	0.34 [0–12]	0.27 [0–10]	- 19.4*	0.07 [0–5]	0.07 [0–4]	- 3.8†	0.07 [0–4]	0.03 [0–4]	- 54.4*	0.20 [0–7]	0.17 [0–7]	- 12.9*
Proportion of patients with appointment, % (±SE)	15.9 (±0.5)	14.3 (±0.5)	-10.0*	5.5 (±0.3)	5.5 (±0.3)	- 0.6	5.4 (±0.3)	2.6 (±0.2)	- 51.5*†	11.0 (±0.4)	10.0 (±0.4)	- 9.0
**Specialised care emergency appointments**												
N of appointments per person, mean [min-max]	0.66 [0–24]	0.69 [0–25]	+ 5.8	0.13 [0–6]	0.15 [0–10]	+ 10.2	0.13 [0–6]	0.12 [0–7]	- 10.2	0.39 [0–14]	0.43 [0–14]	+ 9.8*
Proportion of patients with appointment, % (±SE)	31.4 (±0.6)	31.5 (±0.6)	+ 0.5	9.4 (±0.4)	9.7 (±0.4)	+ 3.5	9.4 (±0.4)	8.3 (±0.4)	- 12.1*	21.4 (±0.5)	22.2 (±0.5)	+ 3.8

*Abbreviations*: *SE* standard error

^1^Face-to-face appointments only in emergency and dental health care

^2^Pre-lockdown periods: 1 January–17 March 2019; 1 January–15 March 2020

^3^Lockdown periods: 18 March–2 June 2019; 16 March–31 May 2020

^4^Post-lockdown periods: 3 June–31 December 2019, 1 June–31 December 2020

* Statistically significant difference between 2019 and 2020 with p-value < 0.05 (Wilcoxon signed-rank test for the difference in continuous variables, logistic regression for proportions)

† Statistically significant difference in the magnitude of change between age group with *p*-value < 0.05 (Mann-Whitney U test for the difference in continuous variables, logistic regression with an interaction term for proportion)

### Emergency visits in specialised care

**Fig. 2:**
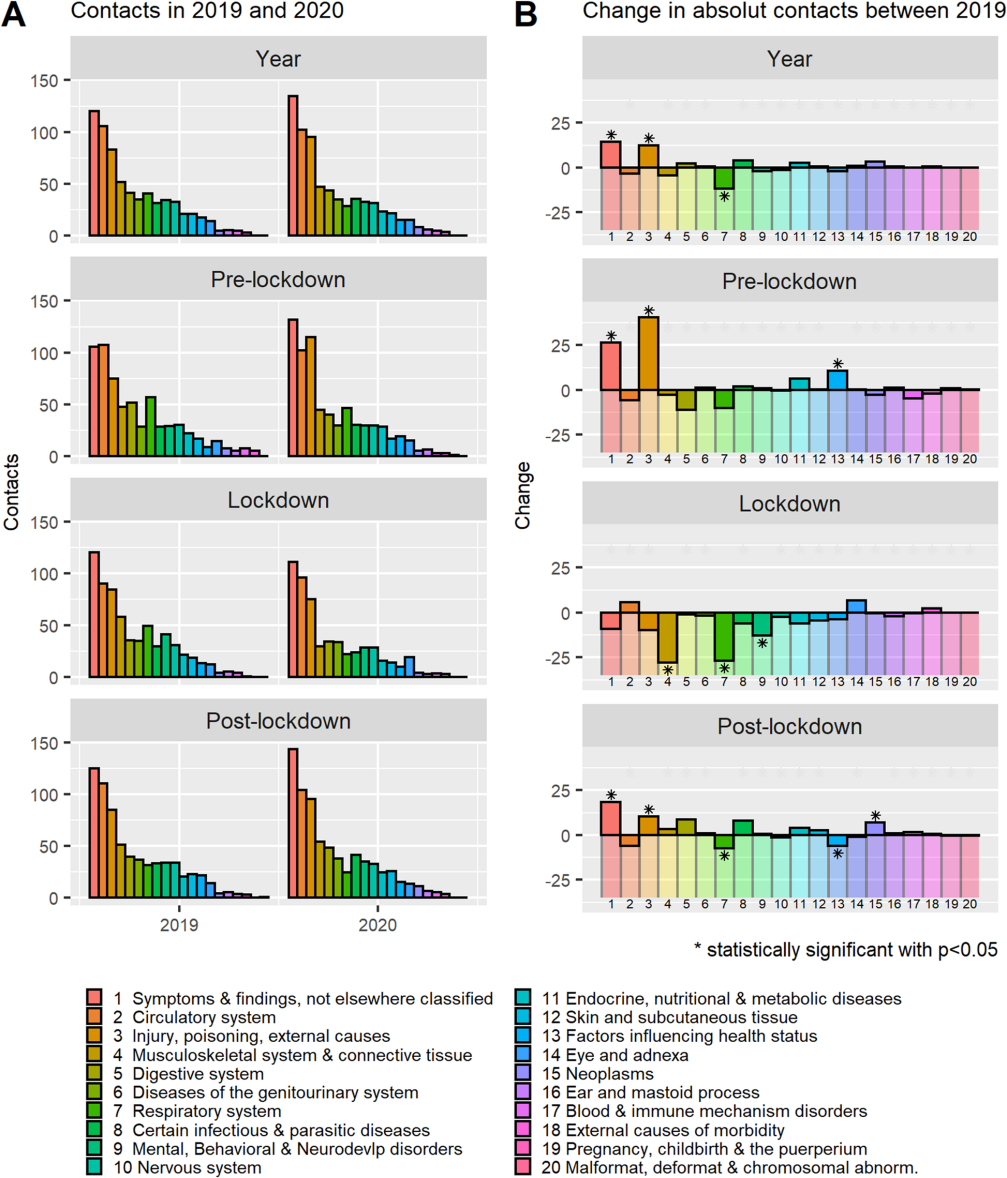
Mean monthly emergency contacts^1^ in 2019 and 2020 and change between the years by CCSR diagnose groups^2^ in different periods^3^. **A**) Mean monthly emergency care contacts by CCSR diagnose groups for 2019 and 2020. **B** Difference between 2019 and 2020 in monthly emergency contacts by CCSR diagnose groups. ^1^The mean monthly visits per period was calculated as the total visits divided by the number of months (12 months for year, 2.5 months for pre-lockdown and lockdown period, 7 months for post-lockdown period). ^2^CCSR categorisation based on ICD-10 diagnosis codes. Some ICD-10 codes are cross-classified into more than one CCSR category because an ICD-10 code can describe multiple conditions or a condition and a common symptom/manifestation. ^3^The periods in 2019 (pre-lockdown period: 1 January to 17 March; lockdown period: 18 March to 2 June; post-lockdown period: 3 June to 31 December) and in 2020 (pre-lockdown period: 1 January to 15 March; lockdown period: 16 March to 31 May; post-lockdown period: 1 June to 31 December). * Statistically significant with *p* < 0.05; *p*-values based on non-parametric Wilcoxon signed-rank test

There was no significant difference in the overall usage of SHC emergency services between the 2 years (*p* = 0.421): about one in four patients sought emergency care (appointments only), and the mean number of appointments was 0.58–0.60 per person (Table 1). While during the lockdown period, emergency care appointments were 14.1% lower (*p* = 0.002) in 2020 than 2019, they were 12.2% (*p* = 0.035) and 5.7% (*p* = 0.147) higher in the pre-lockdown and post lockdown period, respectively. In both years, “symptoms, signs and abnormal clinical and laboratory findings, not classified elsewhere” and “injury, poisoning and other consequences of external causes” belonged to the three most common reasons for an emergency visit and both were statistically significantly higher in the pre-lockdown and post-lockdown period in 2020 than 2019 (Fig. 2). In any period, “Diseases of the respiratory system” were significantly less frequent in 2020 than in 2019, but the decrease was not significant in the pre-lockdown period. A statistically significant decrease in emergency contacts during the lockdown period in 2020 was also observed regarding “diseases of the musculoskeletal system and connective tissue” and “mental, behavioral and neurodevelopmental disorders” compared with the previous year. A list of the 40 most common CCSR categories in either 2019 or 2020 can be found in Supplementary Fig. 1.

### Age differences in healthcare usage

Comparing patients aged < 70 years and ≥ 70 years old, the proportion of patients with appointments dropped significantly more (*p* = 0.048) in 2020 in the older (− 38.8%, *p* < 0.001) than the younger age group (− 34.5%, p < 0.001). Otherwise, annual diabetes care usage in PHC increased or decreased similarly in both age groups regarding the number of overall contacts (p = 0. 535), the proportion of patients with any contact (*p* = 0.133) and remote contacts (*p* = 0.600), and the proportion of remote contacts of all contacts (*p* = 0.736), whereby the proportion of remote consultations increased significantly more (*p* = 0.010) among the older than the younger patient group during lockdown. Older patients had about half as many dental care appointments as younger patients in 2019 (mean 0.34 vs 0.64) and 2020 (0.27 vs 0.54). During the lockdown, the proportion of patients with dental health appointments decreased significantly more (*p* < 0.001) among the older (− 51.5%, p < 0.001) than the younger age group (− 31.6%, p < 0.001). Emergency care services were used more by older than younger patients, but there were no statistically significant age differences regarding the pandemic’s impact. Gender differences in response to the pandemic were also investigated but did not show any significant differences during the lockdown and post-lockdown periods (Supplementary Table 5).

## Discussion

In this EHR-based study, type 2 diabetes patients sought significantly fewer diabetes- and dental health-related PHC services in 2020 than the year before the pandemic, with the most significant reduction during the lockdown period. Diabetes care, which already relied on remote consultations for 55.6% of the contacts during 2019, was realised remotely in 80.0 and 77.1% of the contacts during and after the lockdown, respectively. The high proportions of patients using remote services in both age groups cushioned the drastic decline in face-to-face appointments. Emergency SHC appointments also decreased significantly during the lockdown but quickly increased again so that there was no significant difference when comparing the whole years.

### COVID-19 situation in Finland

From a global perspective, Finland has been in a relatively good situation regarding the pandemic in 2020. The first wave of COVID-19 cases peaked in April with 207 registered new daily cases; the second wave started in September and peaked in November with 619 cases [26]. The Finnish government kept the infection rate down by implementing social distancing measures early with centralised governance [27], refraining from a curfew but closing educational institutions and most government-run public facilities, limiting public gatherings, closing the country’s borders, and implementing a ban on travel in and out of the most affected capital region [18]. Measures in the autumn were less restrictive, allowing regions to react differently based on the local situation [27].

In Finland, municipalities are responsible for organizing and financing health care and most of the health services are public providing residents an equal access to services for free or at reasonable fees. During the lowdown, municipalities and hospital districts were allowed to scale back non-urgent healthcare, but activities expanded again from July onwards when it became clear that there was not an acute threat of overload to the care system [18]. In North Karelia, one of the least affected regions [26], by the end of April people were already warned of the potential health consequences of postponing non-urgent matters and advised to visit the facility in person if the matter could not be handled remotely [28].

### Diabetes care during the pandemic

Our study found significantly fewer diabetes-related PHC contacts in 2020 than the previous year. These findings are consistent with other research, showing that diabetes care and patients’ access to the necessary support to manage their disease and prevent diabetes complications was affected by the pandemic [5–8, 29–31].

In a survey of healthcare professionals from 47 countries, most of the respondents (71.3%) evaluated the management of chronic diseases since the outbreak as not satisfactory, with diabetes being the disease most impacted by the reduction in healthcare resources [7].

Significantly worse results were seen in 288 Catalonian PHC practices regarding indicators relevant to diabetes care during March–April 2020 compared with 2019. For instance, LDL-C and blood pressure control, HbA1c control in type 2 diabetes, and diabetic foot and retinopathy screening activity were significantly lower during the pandemic year [8]. Our study did not report care outcomes.

Forde et al. studied diabetes nurses’ perspectives across Europe and revealed a severe decline in the level of provided diabetes care during the pandemic, reported by 47% of the respondents [6]. The most affected areas included self-management support, diabetes education, and psychological support, reported by 21, 63, and 34% of the respondents, respectively. In a Dutch study of adults with diabetes, many experienced COVID-19-related worries specific to diabetes, including being overly affected if infected (56%), people with diabetes characterised as a risk group (39%), being unable to manage diabetes if infected (28%), and reduced quality of diabetes care (15%) [29]. A study on the early effects of the COVID-19 pandemic on adults with type 1 or type 2 diabetes in the USA found that about every second patient experienced greater difficulty managing their diabetes [30].

Compared to the European average, nurses in Finland less often observed disrupted diabetes-related services (overall diabetes care, self-management, technology and medicines support, and psychological care) and increased physical and psychological risks [6].

Based on a study from fall 2020, in North Karelia, the proportion of patients who did not receive sufficient services from doctors or nurses was 23.0 and 15.5%, respectively [32]. While in Finland there were no major age differences regarding cancelling or postponing services during the early pandemic [21], those aged > 75 years felt good less often about access to health services on the last visit (54.4% vs 58.0–67.9%), less often received examinations and treatments fast enough (54.1% vs 56.2–67.9%) and were more often hampered in their access to care by difficult trips (34.6 vs 26.6–30.7%) than younger adult age groups, respectively [32].

### The role of remote consultations

According to the global survey among healthcare professionals, 35% of the respondents stated that some and 45% stated that all of the appointments were shifted to remote consultations by phone [7]. Mitigation strategies to maintain services during the COVID-19 pandemic included triaging to identify priorities, telemedicine, task shifting/role delegation, novel supply chain or dispensing approaches for medicines through other channels and community outreach to provide information on service disruptions and changes [5].

In Finland, care providers relied already before the pandemic heavily on remote services. In our study, 55.6% of the contacts related to type 2 diabetes happened remotely in 2019. Electronic health records were implemented in North Karelia since 2011 and patients have been able to receive new prescriptions, referrals for examinations, medical opinions, self-care instructions, and lifestyle guidance to monitor and control long-term diseases remotely already for some time [28].

Our study showed that three-quarters of diabetes-related health contacts were conducted remotely in 2020. Recent research from Finland [33, 34] and elsewhere [35–39] has demonstrated the efficacy of telemedicine in type 2 diabetes care, especially teleconsultation [36]. However, reports of the use of remote strategies during the COVID-19 pandemic in other countries are still limited. In Saudi Arabia, newly implemented telemedicine care had significant positive effects on glycaemic control among type 2 diabetes during the COVID-19 pandemic [40]. In a study from Japan, both clinical visits and telemedicine during the early pandemic were independently associated with lower post-emergency HbA1c after adjusting for confounding factors [41]. The situation in Saudi Arabia and Japan, however, differs significantly from our study as remote consultations for diabetes management were implemented during the pandemic for the first time.

### Oral healthcare

We observed a significant drop in PHC dental health visits among type 2 diabetes patients during the lockdown period in 2020, with otherwise quite stable visiting numbers somewhat below the previous year’s levels. Other studies among the general population found a decrease as well in oral health visits during the pandemic. Among the general population in Brazil, PHC oral health visits were 60.7% lower during the first 6 months in 2020 than in 2019 [42]. A study in North Italy, one of the regions the most affected by the pandemic globally in 2020, found an inverse association between the number of urgent dental care visits and the spread of COVID-19 [43]. Similar to our study, visits decreased for women and men regardless of age.

In Finland, a backlog of 1.1 million visits has lengthened queues in primary dental care—especially in areas where the epidemic situation has been most difficult [22]. Comparable with our study, a sharp decrease in visits was observed during lockdown, which then quite rapidly increased again but remained somewhat below the previous year’s levels, which is largely attributable to longer running times than usual due to enhanced protection measures [22]. In addition to the backlog, there are hidden queues of patients who have not yet sought treatment. Depending on the region, it will take one to 3 years to clear the primary oral healthcare backlog [22]. In North Karelia, 25.0% of the patients aged 55–74 years and 20.5% of those aged ≥75 years reported insufficient access to dental services [32]. The most vulnerable individuals, such as type 2 diabetes patients, are at the greatest risk of worsening oral diseases. As a result of delayed access to treatment, oral health problems may have been complicated.

### Emergency care

Our study showed no disruptions to emergency care services among type 2 diabetes patients. According to the second WHO health service continuity survey, emergency care was disrupted to some extent globally in 22% of counties during the last months of 2020 [31]. In contrast with our findings, a major drop in emergency appointments during the early pandemic was observed in studies on the general population in Italy [10, 11], the Netherlands [12], Austria [13], Korea [14], Hong Kong [15] and the USA [16, 17]. Studies covering the whole of 2020 are still lacking, but a timely rebound effect was observed in some studies [16, 17].

Among type 2 diabetes patients, we observed a 14% decrease in emergency visits in 2020 compared to 2019 during the early pandemic phase, while more drastic declines were observed throughout the USA (43%) [16] and in Michigan (48%) [17]. In Michigan, the visits quickly returned to 70% of the previous years’ volume by 31 May.

The absence of children and generally older patients might partially explain the smaller reduction in our study. Studies with age group-specific analyses have found the most significant declines among children, while emergency care usage was less affected among older age groups [10, 12, 14, 16, 17]. In Michigan, emergency care had decreased most to 20% of the levels in 2019 among children (age < 18 years), while it dropped to 55% among patients aged > 70 years [17]. Hartnett et al. (2020) found a sharper decline in emergency care visits among women. Our study did not find any gender- or age-group-specific differences in the change in emergency care usage.

Although emergency care was not substantially impaired, some areas were more affected than others. COVID-19 did not play a major role in emergency care and was diagnosed among only four patients included to this study during 2020. A decrease in COVID-like diagnostic categories (e.g. fever, influenza) was observed in our study as well as elsewhere [16, 17]. We observed a decrease in visits related to “injury, poisoning and other consequences of external causes”, which were significantly more frequent in the pre-lockdown and post-lockdown period but not during the lockdown period in 2020 compared with 2019. Similar to our study, several studies reported a decrease in trauma-related emergency visits during the early pandemic phase [12, 16, 17]. Hartnett et al. (2020) found that the largest reduction in weekly visits during the early pandemic phase were for abdominal pain and other digestive or abdomen signs and symptoms, musculoskeletal pain, and essential hypertension. We also observed a reduction in visits due to essential hypertension, but visits related to abdominal pain and other digestive or abdomen signs and symptoms and musculoskeletal pain were similar or higher when comparing the whole years. Hartnett et al. found a decrease in neoplasm-related encounters during the early pandemic, while we did not observe any difference during the lockdown period, but we did see a statically significant increase in the post-lockdown period. In contrast with Keyes et al. (2021), an increase in patients with alcohol use disorders was not observed in our study. Significant drops were observed regarding acute coronary syndrome [13], acute myocardial Infarction [14], and ST-segment-elevation myocardial infarction [15] during the early pandemic. We observed an increase in visits related to heart failure, but on an aggregated level, “diseases of the circulatory system” did not significantly differ between the years during any of the three periods.

### Strengths and limitations

To our knowledge, this is the first study investigating essential healthcare usage by type 2 diabetes patients in Finland during the pandemic using EHRs covering all PHC and SHC contacts. The strength of the study lies in the analysis of the pandemic’s impact beyond the first months until the end of 2020, providing important insights into the pandemics’ early effects during the lockdown period and the health systems’ adaptation period after the first months of the pandemic. The use of EHRs has the benefit of avoiding non-response, and selection bias as all patients using PHC and SHC are included without requiring the patient’s explicit permission. However, some types of selection bias, such as missing data and patients who visit private care only, cannot entirely be ruled out. Our study also has some limitations. Firstly, despite the increase in video consultations during the pandemic, it was not possible to distinguish between video consultations and phone calls as there were no clear guidelines in place on how to record remote consultations other than as phone calls. Secondly, emergency contacts could be identified reliably for SHC only. In PHC, routine appointments may be booked to a timeslot reserved for urgent matters if there is a time available shortly before. Additionally, our sample was too small to evaluate changes in single diagnoses in different periods.

## Conclusion

Although the COVID-19 pandemic has seriously affected healthcare service usage in North Karelia, essential care was continuously provided. After a drop in SHC emergency visits during the lockdown period, a quick rebound-effect was observed, resulting in similar annual service volumes than 2019. Besides the frequency of healthcare visits, also the composition of emergency usage has not dramatically changed. A drop during the early pandemic phase with subsequently rapidly increasing visits was also observed in PHC contacts related to type 2 diabetes and dental health, although they did not fully reach the previous year’s levels. It was particularly advantageous that the system had already relied heavily on telemedicine for the long-term management of diabetes patients. However, our study also shows that the system has not yet fully recovered. The accumulated and possibly aggravated care demand may soon result in increased health service usage once the COVID-19 vaccination becomes widespread. More research is needed to evaluate the effects of the pandemic on care processes and outcomes.

## Supplementary Information

**Additional file 1: Supplementary Table 1**. *P*-values belonging to Table 1 (“The number of patients and contacts (appointments and remote consultations)”).

**Additional file 2: Supplementary Table 2**. *P*-values belonging to Table 2 (“The number of patients and contacts (appointments and remote consultations) by age group”).

**Additional file 3: Supplementary Table 3**. *P*-values belonging to Supplementary Table 5 (“The number of patients and contacts (appointments and remote consultations) by gender”).

**Additional file 4: Supplementary Table 4** The mean number of contacts per patient (among patients with at least one contact).

**Additional file 5: Supplementary Table 5** The number of patients and contacts (appointments and remote consultations) by gender.

**Additional file 6: Supplementary Figure 1**. Forty most common diagnoses by CCSR for emergency contacts in specialised care either 2019 or 2020 and difference between the years.

## Data Availability

The datasets generated and/or analyzed during the current study are not publicly available due to individual privacy (Personal Data Act) but are available from the corresponding author on reasonable request.

## Abbreviations

CCSR: Clinical Classifications Software Refined
EHR: Electronic health records
ICD-10: International Classification of Diseases (10th revision)
ICPC-2: International Classification of Primary Care (2nd version)
NCD: Non-communicable disease
PHC: Primary healthcare
SHC: Specialised healthcare
