# Supplementary material for: The adverse effect of the COVID-19 pandemic on health service usage among patients with type 2 diabetes in North Karelia, Finland
Source: BMC Health Serv Res. 2022 Jun 1;22:725. doi: 10.1186/s12913-022-08105-z (PMC9156619; doi:10.1186/s12913-022-08105-z)
Supplement: Supplementary file 1 — Additional file 1: Supplementary Table 1. P-values belonging to Table 1 (“The number of patients and contacts (appointments and remote consultations)”). [file 12913_2022_8105_MOESM1_ESM.docx]

**Supplementary Table 1 P-values belonging to Table 1 (“The number of patients and contacts (appointments and remote consultations)”)**

|  |  | **P-values for time difference (2019 vs 2020)^1^** | | | | |
| --- | --- | --- | --- | --- | --- | --- |
|  |  | **Annual** | **Pre- lockdown** | **Lockdown** | | **Post-lockdown** |
| **All patients** |  |  |  |  | |  |
| **Primary care T2D-related contacts (nurse/doctor)** |  |  |  |  |  | |
| N of contacts, N |  | <0.001 | 0.331 | <0.001 | | <0.001 |
| N of contacts per person, mean |  | <0.001 | 0.331 | <0.001 | | <0.001 |
| N of appointments per person, mean |  | <0.001 | 0.001 | <0.001 | | <0.001 |
| N of remote contact per person, mean |  | <0.001 | 0.156 | <0.001 | | <0.001 |
| Proportion of patients with any contact, % |  | <0.001 | 0.043 | <0.001 | | <0.001 |
| Proportion of patients with appointments, % |  | <0.001 | 0.005 | <0.001 | | <0.001 |
| Proportion of patients with remote contact, % |  | <0.001 | 0.339 | <0.001 | | <0.001 |
| Proportion of remote contacts among all contacts, % | | <0.001 | <0.001 | <0.001 | | <0.001 |
| **Primary care T2D-related contacts with nurse** |  |  |  |  | |  |
| N of contacts per person, mean |  | <0.001 | 0.349 | <0.001 | | <0.001 |
| Proportion of patients with any contact, % |  | <0.001 | 0.458 | <0.001 | | <0.001 |
| Proportion of remote contacts among all contacts, % | | <0.001 | 0.086 | <0.001 | | <0.001 |
| **Primary care T2D-related contacts with doctor** |  |  |  |  | |  |
| N of contacts per person, mean |  | <0.001 | 0.281 | <0.001 | | <0.001 |
| Proportion of patients with any contact, % |  | <0.001 | 0.184 | <0.001 | | <0.001 |
| Proportion of remote contacts among all contacts, % | | <0.001 | <0.001 | <0.001 | | <0.001 |
| **Primary care dental health appointments with dentists** |  |  |  |  | |  |
| N of appointments, N |  | <0.001 | <0.001 | <0.001 | | 0.009 |
| N of appointments per person, mean |  | <0.001 | <0.001 | <0.001 | | 0.009 |
| Proportion of patients with appointment, % |  | <0.001 | 0.022 | <0.001 | | 0.045 |
| **Specialised care emergency appointments** |  |  |  |  | |  |
| N of appointments, N |  | 0.421 | 0.035 | 0.002 | | 0.149 |
| N of appointments per person, mean |  | 0.421 | 0.035 | 0.002 | | 0.149 |
| Proportion of patients with appointment, % |  | 0.678 | 0.214 | <0.001 | | 0.672 |

^1^Wilcoxon signed-rank test for the difference in continuous variables, logistic regression for proportion
