# Supplementary material for: The adverse effect of the COVID-19 pandemic on health service usage among patients with type 2 diabetes in North Karelia, Finland
Source: BMC Health Serv Res. 2022 Jun 1;22:725. doi: 10.1186/s12913-022-08105-z (PMC9156619; doi:10.1186/s12913-022-08105-z)
Supplement: Supplementary file 2 — Additional file 2: Supplementary Table 2. P-values belonging to Table 2 (“The number of patients and contacts (appointments and remote consultations) by age group”). [file 12913_2022_8105_MOESM2_ESM.docx]

**Supplementary Table 2 P-values belonging to Table 2 (“The number of patients and contacts (appointments and remote consultations) by age group”)**

|  |  | **P-values for time difference (2019 vs 2020)^1^** | | | |  | **P-values for age difference in change^2^** | | | |
| --- | --- | --- | --- | --- | --- | --- | --- | --- | --- | --- |
| **Age group** |  | **Annual** | **Pre- lockdown** | **Lockdown** | **Post-lockdown** |  | **Annual** | **Pre- lockdown** | **Lockdown** | **Post-lockdown** |
| **Under 70 years (n=5435)** |  |  |  |  |  |  |  |  |  |  |
| **Primary care T2D-related contacts (nurse/doctor)** |  |  |  |  |  |  |  |  |  |  |
| N of contacts, mean [min-max] |  | <0.001 | 0.749 | <0.001 | <0.001 |  | 0.535 | 0.975 | 0.143 | 0.484 |
| Proportion of patients with any contact, % (±SE) |  | <0.001 | 0.058 | <0.001 | <0.001 |  | 0.133 | 0.489 | 0.073 | 0.394 |
| Proportion of patients with appointments, % (±SE) |  | <0.001 | 0.251 | <0.001 | <0.001 |  | 0.048 | 0.267 | <0.001 | 0.362 |
| Proportion of patients with remote contact, % (±SE) |  | <0.001 | 0.711 | <0.001 | <0.001 |  | 0.600 | 0.154 | 0.652 | 0.631 |
| Proportion of remote contacts among all contacts, % (±SE) | | <0.001 | 0.012 | <0.001 | <0.001 |  | 0.736 | 0.849 | 0.010 | 0.631 |
| **Primary care oral health appointments with dentists** |  |  |  |  |  |  |  |  |  |  |
| N of appointments per person, mean [min-max] |  | <0.001 | <0.001 | <0.001 | 0.141 |  | 0.059 | 0.004 | 0.447 | 0.925 |
| Proportion of patients with appointment, % (±SE) |  | <0.001 | 0.004 | <0.001 | 0.252 |  | 0.565 | 0.080 | 0.005 | 0.529 |
| **Specialised care emergency appointments** |  |  |  |  |  |  |  |  |  |  |
| N of appointments per person, mean [min-max] |  | 0.624 | 0.118 | 0.006 | 0.734 |  | 0.104 | 0.959 | 0.645 | 0.083 |
| Proportion of patients with appointment, % (±SE) |  | 0.385 | 0.230 | 0.001 | 0.523 |  | 0.431 | 0.596 | 0.226 | 0.238 |
| **70+ years (n=6023)** |  |  |  |  |  |  |  |  |  |  |
| **Primary care T2D-related contacts (nurse/doctor)** |  |  |  |  |  |  |  |  |  |  |
| N of contacts, mean [min-max] |  | <0.001 | 0.295 | <0.001 | <0.001 |  |  |  |  |  |
| Proportion of patients with any contact, % (±SE) |  | <0.001 | 0.322 | <0.001 | <0.001 |  |  |  |  |  |
| Proportion of patients with appointments, % (±SE) |  | <0.001 | 0.006 | <0.001 | <0.001 |  |  |  |  |  |
| Proportion of patients with remote contact, % (±SE) |  | <0.001 | 0.094 | <0.001 | <0.001 |  |  |  |  |  |
| Proportion of remote contacts among all contacts, % (±SE) | | <0.001 | 0.005 | <0.001 | 0.001 |  |  |  |  |  |
| **Primary care oral health appointments with dentists** |  |  |  |  |  |  |  |  |  |  |
| N of appointments per person, mean [min-max] |  | <0.001 | 0.611 | <0.001 | 0.018 |  |  |  |  |  |
| Proportion of patients with appointment, % (±SE) |  | 0.015 | 0.936 | <0.001 | 0.075 |  |  |  |  |  |
| **Specialised care emergency appointments** |  |  |  |  |  |  |  |  |  |  |
| N of appointments per person, mean [min-max] |  | 0.160 | 0.145 | 0.065 | 0.036 |  |  |  |  |  |
| Proportion of patients with appointment, % (±SE) |  | 0.844 | 0.535 | 0.027 | 0.280 |  |  |  |  |  |

^1^Wilcoxon signed-rank test for the difference in continuous variables, logistic regression for proportions
^2^Mann-Whitney U test for the difference in continuous variables, logistic regression with an interaction term for proportions
