# Supplementary material for: The adverse effect of the COVID-19 pandemic on health service usage among patients with type 2 diabetes in North Karelia, Finland
Source: BMC Health Serv Res. 2022 Jun 1;22:725. doi: 10.1186/s12913-022-08105-z (PMC9156619; doi:10.1186/s12913-022-08105-z)
Supplement: Supplementary file 3 — Additional file 3: Supplementary Table 3. P-values belonging to Supplementary Table 5 (“The number of patients and contacts (appointments and remote consultations) by gender”). [file 12913_2022_8105_MOESM3_ESM.docx]

**Supplementary Table 3 P-values belonging to Supplementary Table 4 (“The number of patients and contacts (appointments and remote consultations) by gender”)**

|  |  |  |  |  |  |  |  |  |  |  |
| --- | --- | --- | --- | --- | --- | --- | --- | --- | --- | --- |
|  |  | **P-values for time difference (2019 vs 2020)^1^** | | | |  | **p-values for gender difference in change^2^** | | | |
| **Gender** |  | **Annual** | **Pre- lockdown** | **Lockdown** | **Post-lockdown** |  | **Annual** | **Pre- lockdown** | **Lockdown** | **Post-lockdown** |
| **Women (n=5223)** |  |  |  |  |  |  |  |  |  |  |
| **Primary care T2D-related contacts (nurse/doctor)** |  |  |  |  |  |  |  |  |  |  |
| N of contacts, mean [min-max] |  | <0.001 | 0.181 | <0.001 | <0.001 |  | 0.495 | 0.325 | 0.794 | 0.088 |
| Proportion of patients with any contact, % (±SE) |  | <0.001 | 0.054 | <0.001 | <0.001 |  | 0.592 | 0.448 | 0.706 | 0.579 |
| Proportion of patients with appointments, % (±SE) |  | <0.001 | 0.016 | <0.001 | <0.001 |  | 0.237 | 0.454 | 0.051 | 0.316 |
| Proportion of patients with remote contact, % (±SE) |  | <0.001 | 0.802 | <0.001 | <0.001 |  | 0.822 | 0.219 | 0.626 | 0.332 |
| Proportion of remote contacts among all contacts, % (±SE) | | <0.001 | 0.010 | <0.001 | <0.001 |  | 0.017 | 0.946 | 0.090 | 0.631 |
| **Primary care oral health appointments with dentists** |  |  |  |  |  |  |  |  |  |  |
| N of appointments per person, mean [min-max] |  | <0.001 | 0.040 | <0.001 | 0.023 |  | 0.614 | 0.717 | 0.467 | 0.445 |
| Proportion of patients with appointment, % (±SE) |  | 0.001 | 0.061 | <0.001 | 0.093 |  | 0.510 | 0.615 | 0.180 | 0.647 |
| **Specialised care emergency appointments** |  |  |  |  |  |  |  |  |  |  |
| N of appointments per person, mean [min-max] |  | 0.575 | 0.517 | 0.001 | 0.699 |  | 0.204 | 0.765 | 0.104 | 0.511 |
| Proportion of patients with appointment, % (±SE) |  | 0.279 | 0.533 | <0.001 | 0.844 |  | 0.273 | 0.748 | 0.146 | 0.501 |
| **Men (n=6234)** |  |  |  |  |  |  |  |  |  |  |
| **Primary care T2D-related contacts (nurse/doctor)** |  |  |  |  |  |  |  |  |  |  |
| N of contacts, mean [min-max] |  | <0.001 | 0.903 | <0.001 | <0.001 |  |  |  |  |  |
| Proportion of patients with any contact, % (±SE) |  | <0.001 | 0.329 | <0.001 | <0.001 |  |  |  |  |  |
| Proportion of patients with appointments, % (±SE) |  | <0.001 | 0.111 | <0.001 | <0.001 |  |  |  |  |  |
| Proportion of patients with remote contact, % (±SE) |  | <0.001 | 0.124 | <0.001 | 0.002 |  |  |  |  |  |
| Proportion of remote contacts among all contacts, % (±SE) | | <0.001 | 0.005 | <0.001 | <0.001 |  |  |  |  |  |
| **Primary care oral health appointments with dentists** |  |  |  |  |  |  |  |  |  |  |
| N of appointments per person, mean [min-max] |  | <0.001 | 0.002 | <0.001 | 0.133 |  |  |  |  |  |
| Proportion of patients with appointment, % (±SE) |  | 0.005 | 0.157 | <0.001 | 0.233 |  |  |  |  |  |
| **Specialised care emergency appointments** |  |  |  |  |  |  |  |  |  |  |
| N of appointments per person, mean [min-max] |  | 0.102 | 0.022 | 0.200 | 0.104 |  |  |  |  |  |
| Proportion of patients with appointment, % (±SE) |  | 0.655 | 0.262 | 0.083 | 0.441 |  |  |  |  |  |

^1^Wilcoxon signed-rank test for the difference in continuous variables, logistic regression for proportions
^2^Mann-Whitney U test for the difference in continuous variables, logistic regression with an interaction term for proportions
