# Supplementary material for: The adverse effect of the COVID-19 pandemic on health service usage among patients with type 2 diabetes in North Karelia, Finland
Source: BMC Health Serv Res. 2022 Jun 1;22:725. doi: 10.1186/s12913-022-08105-z (PMC9156619; doi:10.1186/s12913-022-08105-z)
Supplement: Supplementary file 4 — Additional file 4: Supplementary Table 4 The mean number of contacts per patient (among patients with at least one contact). [file 12913_2022_8105_MOESM4_ESM.docx]

**Supplementary Table 4 The mean number of contacts per patient (among patients with at least one contact)**

|  |  | **Year** | |  | **Pre-lockdown** | |  | **Lockdown** | |  | **Post-lockdown** | |
| --- | --- | --- | --- | --- | --- | --- | --- | --- | --- | --- | --- | --- |
|  |  | **2019** | **2020** |  | **2019** | **2020** |  | **2019** | **2020** |  | **2019** | **2020** |
| **All patients** |  |  |  |  |  |  |  |  |  |  |  |  |
| **Primary care T2D-related contacts (nurse/doctor)** | |  |  |  |  |  |  |  |  |  |  |  |
| N of contacts per patient*, mean |  | 3.48 | 3.47 |  | 1.73 | 1.78 |  | 1.75 | 1.83 |  | 2.48 | 2.58 |
| N of appointments per patient**, mean |  | 1.92 | 1.54 |  | 1.19 | 1.17 |  | 1.19 | 1.06 |  | 1.47 | 1.3 |
| N of remote contacts per patient***, mean |  | 2.63 | 2.98 |  | 1.56 | 1.59 |  | 1.57 | 1.78 |  | 2.05 | 2.35 |
| **Primary care T2D-related contacts with nurse** |  |  |  |  |  |  |  |  |  |  |  |  |
| N of contacts per patient*, mean |  | 2.85 | 2.92 |  | 1.54 | 1.56 |  | 1.52 | 1.66 |  | 2.10 | 2.21 |
| N of appointments per patient**, mean |  | 1.72 | 1.43 |  | 1.15 | 1.13 |  | 1.14 | 1.06 |  | 1.36 | 1.23 |
| N of remote contacts per patient***, mean |  | 2.30 | 2.63 |  | 1.42 | 1.48 |  | 1.42 | 1.63 |  | 1.84 | 2.1 |
| **Primary care T2D-related contacts with doctor** |  |  |  |  |  |  |  |  |  |  |  |  |
| N of contacts per patient*, mean |  | 1.60 | 1.60 |  | 1.16 | 1.17 |  | 1.19 | 1.17 |  | 1.39 | 1.43 |
| N of appointments per patient**, mean |  | 1.10 | 1.10 |  | 1.01 | 1.03 |  | 1.03 | 1.01 |  | 1.06 | 1.05 |
| N of remote contacts per patient***, mean |  | 1.53 | 1.53 |  | 1.14 | 1.15 |  | 1.17 | 1.16 |  | 1.36 | 1.39 |
| **Primary care T2D-related contacts with dentist** |  |  |  |  |  |  |  |  |  |  |  |  |
| N of appointments per patient**, mean |  | 2.26 | 2.11 |  | 1.35 | 1.25 |  | 1.29 | 1.29 |  | 1.86 | 1.82 |
| **By age-group** |  |  |  |  |  |  |  |  |  |  |  |  |
| **Primary care T2D-related contacts (nurse/doctor)** | |  |  |  |  |  |  |  |  |  |  |  |
| **Under 70 years (n=5 435)** |  |  |  |  |  |  |  |  |  |  |  |  |
| N of contacts per patient*, mean |  | 3.57 | 3.58 |  | 1.75 | 1.86 |  | 1.80 | 1.89 |  | 2.56 | 2.67 |
| N of appointments per patient**, mean |  | 1.94 | 1.54 |  | 1.21 | 1.17 |  | 1.20 | 1.06 |  | 1.51 | 1.3 |
| N of remote contacts per patient***, mean |  | 2.70 | 3.10 |  | 1.54 | 1.66 |  | 1.61 | 1.85 |  | 2.12 | 2.44 |
| **70+ years (n=6 023)** |  |  |  |  |  |  |  |  |  |  |  |  |
| N of contacts per patient*, mean |  | 3.4 | 3.37 |  | 1.71 | 1.71 |  | 1.71 | 1.78 |  | 2.41 | 2.49 |
| N of appointments per patient**, mean |  | 1.91 | 1.54 |  | 1.17 | 1.16 |  | 1.18 | 1.07 |  | 1.45 | 1.29 |
| N of remote contacts per patient***, mean |  | 2.57 | 2.88 |  | 1.57 | 1.52 |  | 1.54 | 1.72 |  | 2.00 | 2.27 |
| *among patients with at least one contact |  |  |  |  |  |  |  |  |  |  |  |  |
| ** among patients with at least one appointment |  |  |  |  |  |  |  |  |  |  |  |  |
| ***among patients with at least one remote contact | |  |  |  |  |  |  |  |  |  |  |  |
