# Supplementary material for: The adverse effect of the COVID-19 pandemic on health service usage among patients with type 2 diabetes in North Karelia, Finland
Source: BMC Health Serv Res. 2022 Jun 1;22:725. doi: 10.1186/s12913-022-08105-z (PMC9156619; doi:10.1186/s12913-022-08105-z)
Supplement: Supplementary file 5 — Additional file 5: Supplementary Table 5 The number of patients and contacts (appointments and remote consultations) by gender. [file 12913_2022_8105_MOESM5_ESM.docx]

**Supplementary Table 5 The number of patients and contacts (appointments and remote consultations)^1^ by gender**

|  |  |  |  |  |  |  |  |  |  |  |  |  |  |  |  |  |
| --- | --- | --- | --- | --- | --- | --- | --- | --- | --- | --- | --- | --- | --- | --- | --- | --- |
|  |  | **Year** | | |  | **Pre-lockdown^2^** | | |  | **Lockdown^3^** | | |  | **Post-lockdown^4^** | | |
|  |  | 2019 | 2020 | Change (in %) |  | 2019 | 2020 | Change (in %) |  | 2019 | 2020 | Change (in %) |  | 2019 | 2020 | Change (in %) |
| **Women (n=5223)** |  |  |  |  |  |  |  |  |  |  |  |  |  |  |  |  |
| **Primary care T2D-related contacts (nurse/doctor)** |  |  |  |  |  |  |  |  |  |  |  |  |  |  |  |  |
| N of contacts, N [min-max] |  | 2.32 [0-42] | 2.13 [0-33] | -8.1* |  | 0.5 [0-11] | 0.49 [0-11] | -2.3 |  | 0.54 [0-17] | 0.45 [0-13] | -15.7* |  | 1.28 [0-26] | 1.19 [0-26] | -7.2* |
| Proportion of patients with any contact, % (±SE) |  | 66.3 (±0.7) | 60.9 (±0.7) | -8.2* |  | 29.4 (±0.6) | 27.7 (±0.6) | -5.8 |  | 30.7 (±0.6) | 25.1 (±0.6) | -18.4* |  | 51.8 (±0.7) | 45.5 (±0.7) | -12.0* |
| Proportion of patients with appointments, % (±SE) |  | 51.9 (±0.7) | 31.8 (±0.6) | -38.7* |  | 17.7 (±0.5) | 15.9 (±0.5) | -9.9* |  | 19.9 (±0.6) | 4.5 (±0.3) | -77.4* |  | 37.6 (±0.7) | 19.4 (±0.5) | -48.4* |
| Proportion of patients with remote contact, % (±SE) |  | 50.1 (±0.7) | 54.0 (±0.7) | +7.7* |  | 18.9 (±0.5) | 18.7 (±0.5) | -1.0 |  | 19.1 (±0.5) | 23.2 (±0.6) | +21.1* |  | 35.3 (±0.7) | 39.3 (±0.7) | +11.2* |
| Proportion of remote contacts among all contacts, % (±SE) | | 57.2 (±0.4) | 77.4 (±0.4) | +35.3*† |  | 58.4 (±1.0) | 62.0 (±1.0) | +6.2* |  | 56.1 (±0.9) | 89.5 (±0.6) | +59.5* |  | 57.2 (±0.6) | 79.1 (±0.5) | +38.3* |
| **Primary care oral health appointments with dentists** |  |  |  |  |  |  |  |  |  |  |  |  |  |  |  |  |
| N of appointments per person, mean [min-max] |  | 0.45 [0-13] | 0.36 [0-10] | -19.6* |  | 0.10 [0-8] | 0.08 [0-4] | -14.6* |  | 0.09 [0-5] | 0.05 [0-3] | -44.6* |  | 0.26 [0-8] | 0.22 [0-8] | -12.3* |
| Proportion of patients with appointment, % (±SE) |  | 20.7 (±0.6) | 18.0 (±0.5) | -12.7* |  | 7.5 (±0.4) | 6.5 (±0.3) | -12.5 |  | 7.4 (±0.4) | 4.1 (±0.3) | -44.0* |  | 14.3 (±0.5) | 13.1 (±0.5) | -7.9 |
| **Specialised care emergency appointments** |  |  |  |  |  |  |  |  |  |  |  |  |  |  |  |  |
| N of appointments per person, mean [min-max] |  | 0.61 [0-26] | 0.61 [0-24] | -1.2 |  | 0.12 [0-6] | 0.13 [0-10] | +6.3 |  | 0.13 [0-6] | 0.1 [0-7] | -19.5* |  | 0.36 [0-20] | 0.37 [0-15] | +2.8 |
| Proportion of patients with appointment, % (±SE) |  | 29.0 (±0.6) | 28.0 (±0.6) | -3.3 |  | 8.6 (±0.4) | 8.9 (±0.4) | +4.0 |  | 9.0 (±0.4) | 7.0 (±0.4) | -21.7* |  | 19.8 (±0.6) | 19.7 (±0.6) | -0.8 |
| **Men (n=6234)** |  |  |  |  |  |  |  |  |  |  |  |  |  |  |  |  |
| **Primary care T2D-related contacts (nurse/doctor)** |  |  |  |  |  |  |  |  |  |  |  |  |  |  |  |  |
| N of contacts, N [min-max] |  | 2.26 [0-35] | 2.04 [0-28] | -10.1* |  | 0.5 [0-17] | 0.5 [0-17] | -0.5 |  | 0.52 [0-14] | 0.44 [0-12] | -15.6* |  | 1.24 [0-25] | 1.1 [0-22] | -11.6* |
| Proportion of patients with any contact, % (±SE) |  | 65.4 (±0.6) | 59.2 (±0.6) | -9.5* |  | 28.5 (±0.6) | 27.7 (±0.6) | -2.8 |  | 29.7 (±0.6) | 23.8 (±0.5) | -20.0* |  | 50.1 (±0.6) | 43.1 (±0.6) | -13.8* |
| Proportion of patients with appointments, % (±SE) |  | 53.6 (±0.6) | 34.7 (±0.6) | -35.2* |  | 19.0 (±0.5) | 17.9 (±0.5) | -5.8 |  | 20.3 (±0.5) | 5.5 (±0.3) | -72.8* |  | 38.1 (±0.6) | 20.8 (±0.5) | -45.6* |
| Proportion of patients with remote contact, % (±SE) |  | 47.0 (±0.6) | 51.2 (±0.6) | +8.9* |  | 17.5 (±0.5) | 18.6 (±0.5) | +6.0 |  | 17.8 (±0.5) | 21.1 (±0.5) | +18.6* |  | 33.2 (±0.6) | 35.8 (±0.6) | +7.8* |
| Proportion of remote contacts among all contacts, % (±SE) | | 54.2 (±0.4) | 73.4 (±0.4) | +35.4*† |  | 54.6 (±0.9) | 58.1 (±0.9) | +6.4* |  | 53.4 (±0.9) | 86.6 (±0.6) | +62.2* |  | 54.4 (±0.6) | 75.2 (±0.5) | +38.2* |
| **Primary care oral health appointments with dentists** |  |  |  |  |  |  |  |  |  |  |  |  |  |  |  |  |
| N of appointments per person, mean [min-max] |  | 0.51 [0-12] | 0.43 [0-11] | -14.4* |  | 0.12 [0-6] | 0.10 [0-6] | -18.1* |  | 0.10 [0-4] | 0.07 [0-4] | 34.9* |  | 0.28 [0-8] | 0.27 [0-11] | -5.4 |
| Proportion of patients with appointment, % (±SE) |  | 21.7 (±0.5) | 19.6 (±0.5) | -9.4* |  | 8.4 (±0.4) | 7.7 (±0.3) | -8.2 |  | 8.0 (±0.3) | 5.2 (±0.3) | -35.0* |  | 14.9 (±0.5) | 14.2 (±0.4) | -5.1 |
| **Specialised care emergency appointments** |  |  |  |  |  |  |  |  |  |  |  |  |  |  |  |  |
| N of appointments per person, mean [min-max] |  | 0.55 [0-34] | 0.59 [0-54] | +6.8 |  | 0.11 [0-6] | 0.13 [0-15] | +17.8* |  | 0.11 [0-10] | 0.1 [0-13] | -8.9 |  | 0.33 [0-21] | 0.36 [0-32] | +8.4 |
| Proportion of patients with appointment, % (±SE) |  | 26.2 (±0.6) | 26.5 (±0.6) | +1.3 |  | 7.7 (±0.3) | 8.3 (±0.3) | +7.0 |  | 7.6 (±0.3) | 6.8 (±0.3) | -10.5 |  | 17.7 (±0.5) | 18.2 (±0.5) | +3.0 |

^1^Face-to-face appointments only in emergency and oral health care. ^2^Pre-lockdown periods: 1 January–17 March 2019; 1 January–15 March 2020
^3^Lockdown periods: 18 March–2 June 2019; 16 March–31 May 2020
^4^Post-lockdown periods: 3 June–31 December 2019, 1 June–31 December 2020
* Statistically significant difference between 2019 and 2020 with p-value < 0.05 (Wilcoxon signed-rank test for the difference in continuous variables, logistic regression for proportions)
† Statistically significant difference in the magnitude of change between gender with p-value < 0.05 (Mann-Whitney U test for the difference in continuous variables, logistic regression with an interaction term for proportions)
