# Supplementary material for: The adverse effect of the COVID-19 pandemic on health service usage among patients with type 2 diabetes in North Karelia, Finland
Source: BMC Health Serv Res. 2022 Jun 1;22:725. doi: 10.1186/s12913-022-08105-z (PMC9156619; doi:10.1186/s12913-022-08105-z)
Supplement: Supplementary file 6 — Additional file 6: Supplementary Figure 1. Forty most common diagnoses by CCSR for emergency contacts in specialised care either 2019 or 2020 and difference between the years. [file 12913_2022_8105_MOESM6_ESM.docx]

**Supplementary Figure 1 Forty most common diagnoses by CCSR for emergency contacts in specialised care either 2019 or 2020 and difference between the years**


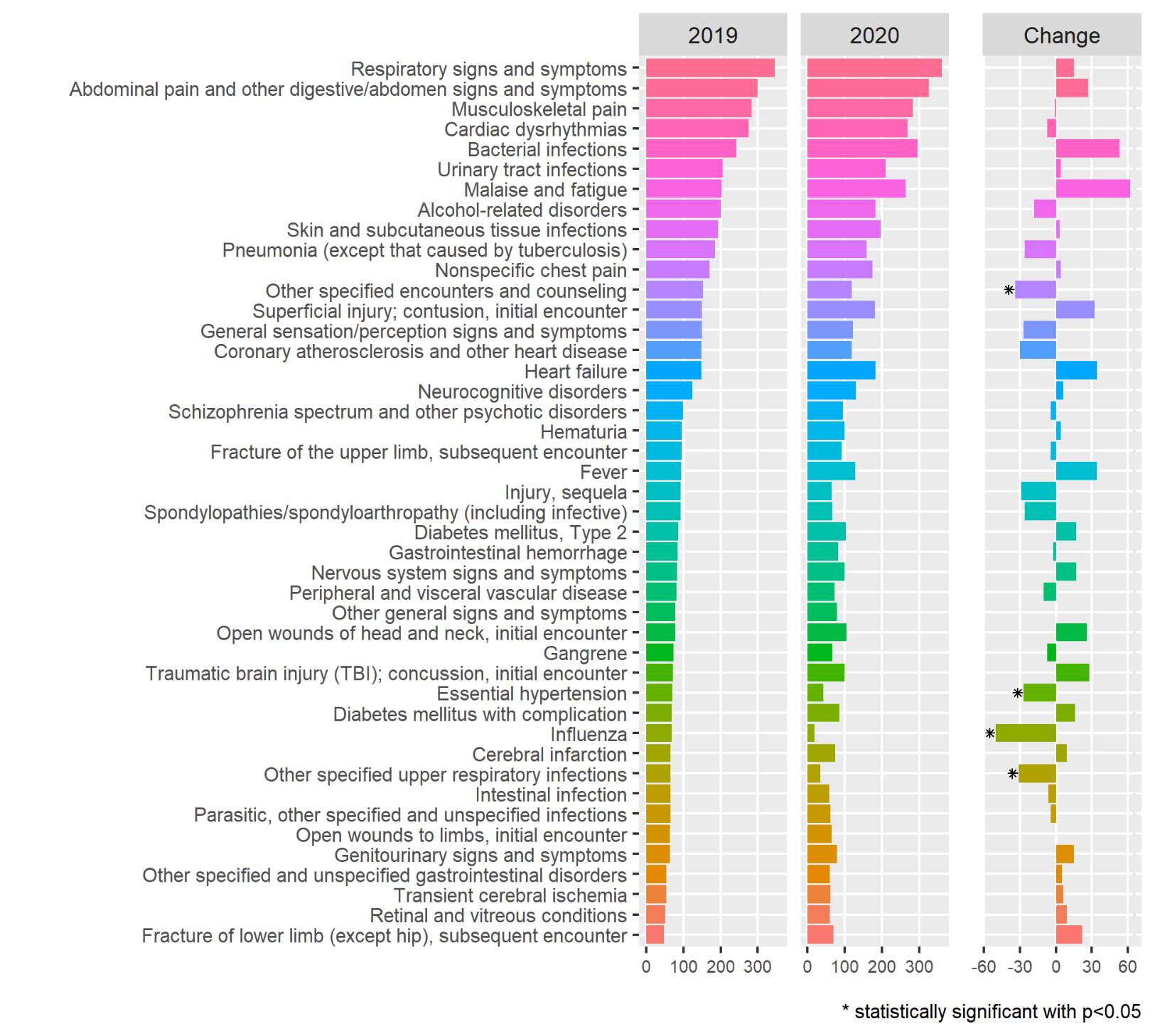


* Statistically significant difference between 2019 and 2020 with p-value < 0.05; Wilcoxon signed-rank test
